# A comparative analysis of gut microbiome in dogs using short- and long-reads of 16S rRNA sequences reveals workflow-dependent biases

**DOI:** 10.1007/s11259-026-11407-w

**Published:** 2026-07-17

**Authors:** Giulia Polacchini, Bruno Stefanon, Paolo Mongillo, Danilo Licastro

**Affiliations:** 1 Department of Agricultural, Food, Environmental and Animal Sciences, University of Udine, Udine, Italy; 2 Department of Comparative Biomedicine and Food Science, University of Padova, Padova, Italy; 3 ARGO Open Lab Platform for Genome Sequencing, AREA Science Park, Trieste, Italy

**Keywords:** Microbiome comparison, Nanopore sequencing, Illumina sequencing, Sequencing workflow comparison, Data comparability

## Abstract

Short-read Illumina sequencing of hypervariable regions of the 16S rRNA gene and long-read Oxford Nanopore Technologies (ONT) sequencing of the full-length 16S gene are increasingly used to profile microbial communities. However, differences in sequencing chemistry, read length, and taxonomic assignment methods raise concerns about the comparability of microbiome profiles and their impact on biological interpretation. Faecal samples from two groups of healthy dogs, 8 young (32 < age < 59 months) and 8 old (age > 109 months), were analysed. Taxonomic relative abundances obtained from Illumina and ONT sequencing were compared after nomenclature harmonisation. Agreement between workflows was assessed using Bland–Altman analysis on log2-transformed relative abundances. Although the mean bias at the genus level was small (0.229), limits of agreement indicated poor interchangeability between workflows. When restricted to taxa detected by both workflows, variability decreased but a larger negative bias emerged, suggesting abundance-dependent discrepancies. The effects of sequencing workflow on biologically relevant signals related to host age were evaluated by computing alpha and beta diversity metrics independently within each workflow. Alpha diversity differed between workflows, with ONT yielding higher Shannon diversity and richness values than Illumina. Beta diversity analyses indicated significant (*p* < 0.05) age-related shifts in community composition only in ONT, with differences in variance explained and effect sizes. Workflow comparisons revealed taxon-specific differences at phylum, family, and genus levels, affecting both moderately and highly abundant taxa. These findings indicate that sequencing workflow choice impacts microbiome profiling and downstream interpretation, underscoring the need for care in cross-workflow comparisons.

**Supplementary Information:**

The online version contains supplementary material available at https://doi.org/10.1007/s11259-026-11407-w.

## Highlights

Illumina and Nanopore show limited interchangeability in microbiome profiling.

Workflow choice significantly alters relative abundance estimates across taxa.

Bias increases at finer taxonomic resolution.

Detection differences drive cross-workflow discrepancies.

Sequencing workflow impacts alpha and beta diversity metrics.

**Supplementary Information:**

The online version contains supplementary material available at https://doi.org/10.1007/s11259-026-11407-w.

## Introduction

The microbiota is usually defined as the totality of living microorganisms in a given environment (Marchesi and Ravel 2015). The microbial populations change throughout the digestive tract, but the major bacterial taxa are consistently present in faecal samples from the healthy host, indicating a bacterial core (Martínez et al. 2013). High-throughput sequencing of the 16S rRNA gene has become the first cost effective choice for characterizing microbial communities of gut among host species, environmental and health conditions. The 16S rRNA gene is highly conserved among bacterial species and consists of approximately 1500 bp encoding a subunit of the ribosome. It contains conserved regions that facilitate the alignment of genes between different bacterial species, but also hypervariable regions that allow differentiation between taxa at the genus or species level.

Over the years, various NGS (Next Generation Sequencing) methods and sequencing platforms have been developed to increase accuracy, decrease error rates, reduce run time and costs, and facilitate computer-assisted analysis of the microbiota, enabling the detection of differences in bacterial composition down to the species level (Di Bella et al. 2013). Different sequencing workflows can produce comparable overall profiles of microbiota composition, but the average relative abundance of specific taxa can vary, as factors such as primer selection for library preparation can influence the detection of genera and species (Allali et al. 2017; Fouhy et al. 2016).

Different hypervariable regions of the 16S rRNA gene provide different levels of taxonomic discrimination across bacterial groups, and no single region performs optimally for all taxa (Hoffbeck et al. 2025; Johnson et al. 2019; Yang et al. 2016). Consequently, microbiome profiles may vary depending not only on sequencing technology but also on the specific region targeted by amplification primers. The commonly used V3–V4 region offers a favorable compromise between taxonomic resolution and sequencing performance for gut microbiome studies, although certain bacterial taxa may be preferentially detected or underrepresented relative to analyses based on other hypervariable regions or full-length 16S sequencing.

Short-read sequencing platforms such as Illumina, targeting hypervariable regions of the 16S rRNA gene (e.g., V3–V4), have dominated microbiome research due to costs and available analytical pipelines. Long-read sequencing technologies, as Oxford Nanopore Technologies (ONT), have emerged as alternatives by enabling sequencing of the full-length 16S rRNA gene, potentially improving taxonomic resolution and assignment (Matsuo et al. 2021). Illumina- and ONT-based microbiome profiling workflows differ in the targeted 16S rRNA gene region, primer design, sequencing chemistry, read length, reference databases, and downstream bioinformatic processing (Abellan-Schneyder et al. 2021). Because both short-read and long-read platforms can be adapted to sequence different portions of the 16S rRNA gene (Burke and Darling 2016), the present comparison should be interpreted as an evaluation of complete microbiome profiling workflows rather than an isolated assessment of sequencing technology. These differences raise an important methodological question: to what extent are microbiota profiles generated by commonly used Illumina and ONT workflows comparable? This issue is particularly relevant when cross-study comparisons, meta-analyses, and biological interpretations are considered. Previous studies have reported that reference database selection alone can substantially influence taxonomic assignments and relative abundance estimates, even when the same sequencing data and analytical pipeline are used (Sierra et al. 2020; Baghbanzadeh et al. 2025).

Researchers have shown that full-length 16S sequencing can improve taxonomic resolution compared with target individual variable regions, particularly at the species level. Johnson et al. (2019) demonstrated that sequencing the entire 16S gene provides greater discriminatory power than short-read regions and can better capture microbial diversity. Other studies (Martijn et al. 2019; Yeo et al. 2024) have evaluated the analytical performance of long-read 16S sequencing, including both PacBio- and ONT-based workflows, and have reported improvements in taxonomic resolution in comparison to short-read sequencing approaches. However, investigations have focused primarily on taxonomic resolution, classification accuracy, or methodological benchmarking. Little attention has been paid to understanding how these methodological differences can influence ecological metrics, biological interpretation, and detection of relevant patterns in microbiome datasets.

In this study, taxonomic relative abundances (RA) obtained from Illumina and ONT sequencing were compared after nomenclature harmonisation to assess the extent workflow-associated differences can affect ecological and biological conclusion. The ability of the two workflows to capture biologically relevant signals associated with host age (young vs. old) was also evaluated by computing diversity metrics independently within each workflow.

## Materials and methods

### Animal ethics statement

In this study, faecal samples from 16 healthy male Beagle dogs were used, representing a subgroup of the 175 dogs included in the study by Balouei et al. (2025), where details are reported. Briefly, dogs had the same standard commercial kibble diet and water was provided ad libitum. The inclusion criteria were without major or minor (within 6 months) pathologies and without vaccinations and parasite treatments for at least 3 months. The protocol was approved by the study facility’s Institutional Animal Care and Use Committee (IACUC) before the start of the trial, as per IACUC standard operating procedures.

### Animals and DNA extraction from faecal samples

Faecal samples of 16 male Beagle dogs were collected. Dogs were divided by age. The group of young dogs ranged in age from 32 to 59 months and the group of old dogs ranged in age from 109 to 146 months. For each individual dog, faecal samples were collected and stored in Zymo Shield (Zymo Research, Irvine, CA, USA) to stabilise DNA, RNA and proteins. Microbial DNA was extracted with the Quick-DNA Fecal/Soil Microbe Miniprep™ kit (Zymo Research, Irvine, CA, USA) according to the manufacturer’s instructions, and the extracted DNA was quantified with the Qubit™ 1X dsDNA Broad Range Assay kit (Invitrogen; Waltham, MA, USA) using the Qubit™ 3 Fluorometer (Thermo Fisher Scientific; Waltham, MA, USA). The purity of samples with concentrations between 5 and 24 ng/µL was assessed using the nucleic acid quantification tool of the Spark microplate reader (Tecan Group Ltd., Switzerland). DNA samples with purity values between 2.0 and 2.2 were sequenced. DNA extracted from the same sample was aliquoted, with one aliquot sequenced using the Illumina workflow and the other using the ONT workflow, thereby minimising pre-analytical variability.

### Illumina sequencing and taxonomic annotation

Illumina sequencing was performed by sequencing providers, who also assessed the quality of the extracted DNA and the libraries.

For each faecal sample, the 16S rRNA V3 and V4 hypervariable regions were amplified for library preparation by PCR (polymerase chain reaction) using primers according to Klindworth et al. (2013) as the most promising bacterial primer pair. The full-length primer sequences were 5’-CCTACGGGNGGCWGCAG-3’ for the forward primer and 5’- GACTACHVGGGTATCTAATCC-3’ for the reverse primer.

The library was prepared using the Nextera XT kit (Illumina; San Diego, CA, USA) and amplicons were purified using Agencourt AMPure XP beads (Beckman Coulter Genomics; Danvers, MA, USA) and a magnetic stand. The expected size of the amplicons was approximately 630 bp. A 10 pM pooled library was sequenced using second generation sequencing technology on the NovaSeq 6000 Illumina platform (Ilumina; San Diego, CA, USA) in 2 × 250 paired-end mode according to standard procedures. Raw sequences in FASTQ format generated by the Illumina were processed with QIIME2 (quantitative insights into microbial ecology 2) v2020.11 bioinformatic tool (Bolyen et al. 2019), using DADA2 v1.18.0 (Callahan et al. 2016), filtering reads with a Phred score greater than or equal to 25. Sequencing was conducted to achieve an expected depth of approximately 50,000 reads per sample. Taxonomic assignment was based on the QIIME2/Greengenes database 2024.10 (greengenes.lbl.gov/).

### Oxford Nanopore sequencing and taxonomic annotation

From each faecal sample, 10 ng of extracted DNA was used for library preparation with the 16S Barcoding Kit 1–24 (Oxford Nanopore Technologies; Oxford, UK) according to manufacturer instructions. The kit amplifies approximately 1.5 kb of the full-length 16S rRNA gene using the universal primers 27F (5’-AGAGTTTGATCMTGGCTCAG-3’) and 1492R (5’-CGGTTACCTTGTTACGACTT-3’) (Graham et al. 2024).

A total of 90 ng of the library was pooled and sequenced using third generation sequencing technology on the MinION™ platform (Oxford Nanopore Technologies; Oxford, UK) using the R9.4.1 SpotON Flow Cell and the integrated 16S Barcoding Kit 1–24 script on MinKNOW software v20.06.17 for 20 h. The script also performed the basecalling, demultiplexing process, chimera removal and filtered reads with a Phred score greater than or equal to 25 immediately after the sequencing run. Sequencing was conducted to achieve an expected depth of approximately 50,000 reads per sample. Nanopore reads were taxonomically classified using the EPI2ME bioinformatics platform (Oxford Nanopore Technologies, Oxford, UK) employing the 16S workflow, which uses the Kraken2 algorithm (v2.17.1, 24.11.2025) for taxonomic assignment against the NCBI reference database (Bertolo et al. 2024), accessed in January 2026.

### Statistical analysis of differential taxa abundance

Feature tables were converted to RA by normalizing counts to the total sequencing depth per sample (Weiss et al. 2017). Taxa were filtered prior to testing to reduce noise and multiple testing burden. A taxon was retained if it was present (RA > 0) in at least 25% of samples in at least one Age group and its mean RA across all samples was ≥ 0.1% (0.001). Taxonomic harmonisation was performed by matching identical taxon names and manually curated synonyms between Greengenes-based Illumina annotations and NCBI-based ONT annotations. At the phylum level, 8 taxa were mapped, whereas 4 Illumina-only taxa remained unmapped. At the family level, 23 taxa were mapped, while 21 ONT-only and 30 Illumina-only taxa remained unmapped. At the genus level, 35 taxa were mapped, whereas 93 ONT-only and 49 Illumina-only genera remained unmapped. These unmatched taxa were not included in direct workflow-level abundance comparisons and are reported in Supplementary Table S1.

To assess systematic differences in taxonomic abundance estimates between sequencing workflows, RA tables derived from Illumina and ONT were compared at phylum, family and genus levels. Comparative analysis was not applied at species level, considering the low reliability of annotation based on short reads. For the aim of this study, data were presented for phylum, family and genus.

Agreement between taxonomic profiles generated by Illumina and ONT was assessed using Bland–Altman analysis on RA, after addition of a pseudocount of 10^− 6^ and log2 transformation. The following quantities were computed:

The mean abundance between workflows:

$$M_i=\lbrack\log2(RA_{ONT,i}+10^{-6})+\log2(RA_{Illumina,i}+^{-6})\rbrack/2$$

The difference between workflows:

$$D_i=\log2(RA_{ONT,i}+10^{-6})-\log2(RA_{Illumina,i}+10^{-6})$$

The mean difference (bias) was calculated as the average of the differences (D_i_) across all observations:

$$\overline D=(1/n)\sum D_i$$

The limits of agreement (LoA) were defined as:

$$\overline D \pm 1.96 \times SD(D)$$

Two Bland–Altman analyses were performed: Using the complete dataset, including all taxon–sample pairs, and using only taxa with non-zero relative abundance in both workflows. Bland–Altman plots were used as an exploratory descriptive approach to visualize agreement and systematic differences between sequencing workflows. Because relative abundances are compositional and not statistically independent, Bland–Altman plots were interpreted as descriptive visualizations of concordance rather than formal assessments of measurement agreement. To visualise taxon-specific differences between workflows, MA plots were generated using median relative abundances for each taxon. MA plots display the relationship between the average abundance of each taxon across workflows (A) and the corresponding abundance difference between workflows (M), allowing identification of taxa showing the largest workflow-dependent deviations. Differences between workflows were assessed using Wilcoxon tests, and *P*-values were adjusted within each taxonomic level using the Benjamini–Hochberg false discovery rate (FDR) procedure.

### Alpha and beta diversity analysis

The comparison of Shannon and Chao1 alpha diversity indexes between old and young dogs at a genus level was assessed separately for Illumina and ONT, using the Wilcoxon rank-sum test with FDR correction. Alpha diversity distributions were visualized using boxplots stratified by sequencing workflows. Community dissimilarities at genus level were computed using the Bray–Curtis distance metric separately for each sequencing workflow and reported in Principal Coordinates Analysis (PCoA). The effect of Age on beta diversity was assessed using permutational multivariate analysis of variance (PERMANOVA) implemented via the adonis2 function v.2.7.3 with 999 permutations.

## Results

### Differences between Illumina and ONT microbiome profiling workflows

**Fig. 1:**
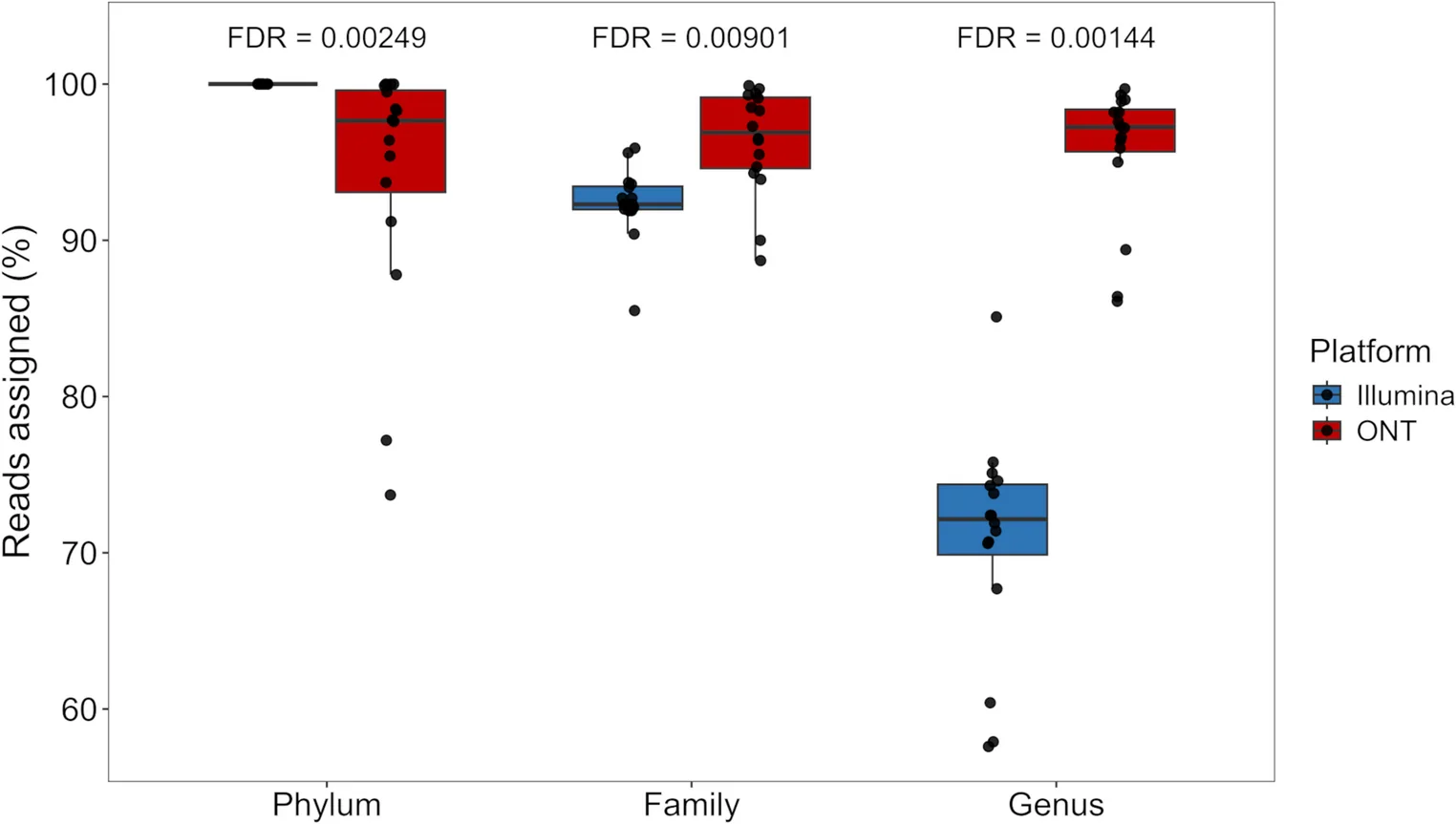
Taxonomic assignment rates at phylum, family, and genus levels for Illumina and ONT sequencing workflows. Boxes represent the distribution of the percentage of reads assigned across samples. FDR-adjusted *p*-values are reported for workflow comparisons within each taxonomic level

The proportion of taxonomically assigned reads differed significantly between sequencing workflows. At the phylum level, Illumina and ONT showed significant difference (FDR = 0.00249) in RA assignment value. ONT sequencing yielded higher proportions of taxonomically assigned reads, with a median of 96.9% of reads (FDR = 0.0091) assigned at the family level and 97.3% (FDR = 0.00144) at the genus level (Fig. 1). In contrast, Illumina sequencing showed lower performance, with 92.3% and 72.1% of reads assigned at a family and genus levels, respectively.

**Fig. 2:**
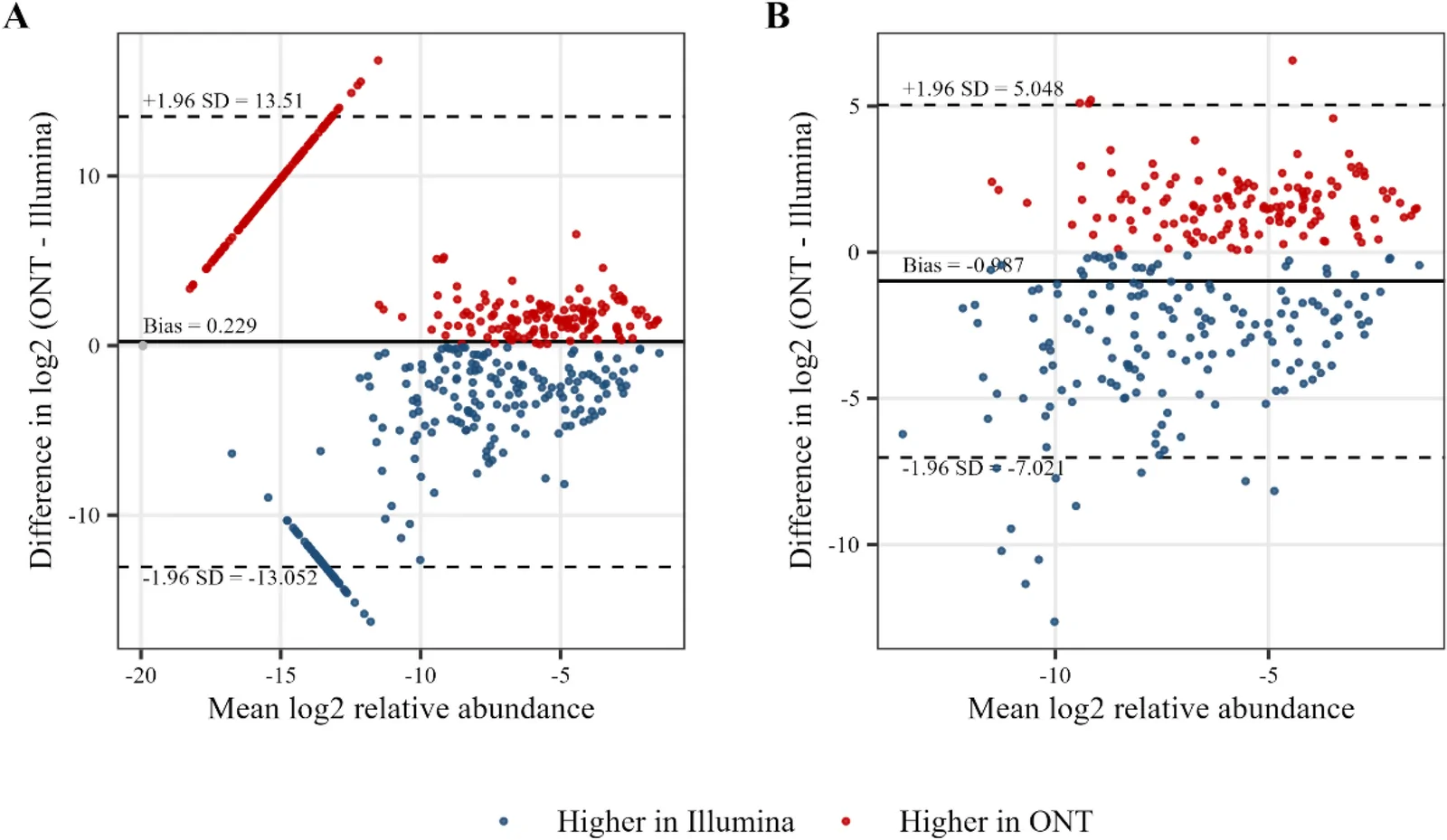
Bland–Altman concordance analysis between Illumina and Oxford Nanopore (ONT) sequencing workflows at genus level. Panel **A** shows the full dataset, where diagonal patterns arise from taxa detected in only one workflow, indicating a substantial discordance in genus detection for low-abundance taxa. Panel **B** shows when the analysis is restricted to taxa with non-zero relative abundance in both workflows, the dispersion of differences being markedly reduced and the limits of agreement became narrower

The Bland–Altman analysis was employed to evaluate bias and limits of agreement in log_2_ transformed RA. This approach allows identification of workflow-dependent distortions and highlights differences associated with low-abundance taxa. The Bland–Altman analysis performed on the complete genus-level dataset (560 paired observations) showed a negligible mean bias (< 0.001 log2 relative abundance units), with limits of agreement ranging from − 0.162 to 0.162 log2 units (Fig. 2A). The same analysis was also conducted at a phylum and family taxonomic levels (Figure S1A and S1B). The characteristic diagonal patterns observed in these Bland–Altman plots arose from taxa detected by only one workflow, reflecting detection discordance rather than purely quantitative disagreement. The mean bias at the genus level was 0.229 log2 units, corresponding to an average difference of approximately 17% in relative abundance estimates between workflows. However, the limits of agreement were broad (− 13.05 to 13.51 log2 units), indicating that abundance estimates for individual taxa could differ by several orders of magnitude, thus limiting the interchangeability of Illumina and ONT for quantitative comparisons. This suggests that, despite the absence of a consistent directional bias, substantial variability exists between workflows. When the analysis was restricted to taxa with non-zero relative abundance in both workflows at genus level (Fig. 2B) and at phylum and family levels (Figure S1C and S1D), the dispersion of differences was markedly reduced and the limits of agreement became narrower, but the variability between workflows was higher (mean bias − 0.987 for genus taxa, limits of agreement upper 5.048, lower − 7.021), indicating that differences are not exclusively due to detection thresholds. To further describe taxon detectability across workflows, median relative abundance, range, and prevalence values for family- and genus-level taxa included in the comparative analyses are reported in Supplementary Table S3. These summaries show that several taxa were detected at low prevalence or only in one workflow, supporting the interpretation that part of the observed disagreement was driven by presence/absence discordance among low-abundance taxa.

**Fig. 3:**
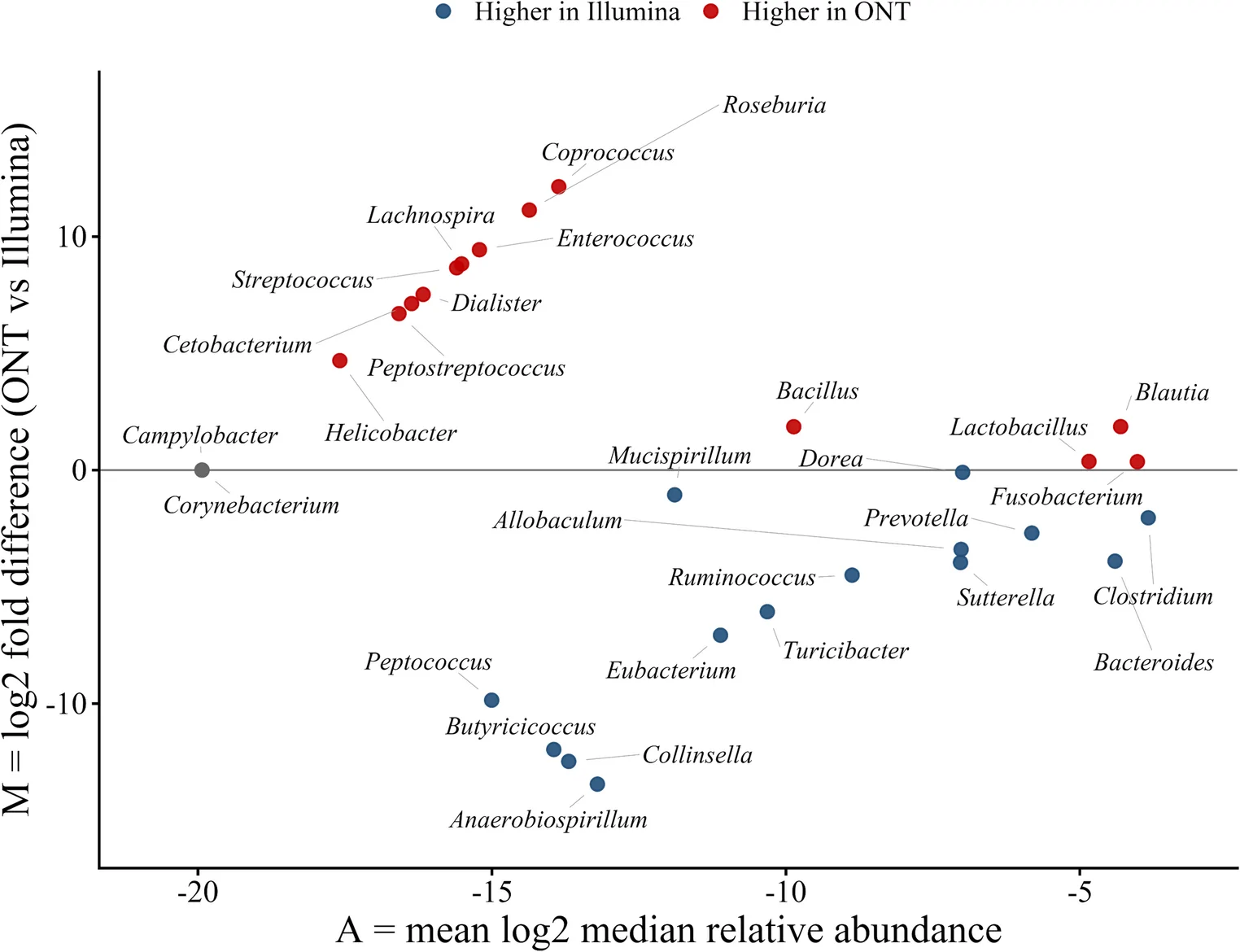
MA plot of significant taxa at Genus level comparing Illumina and ONT. The x-axis shows mean relative abundance (A, log2 scale), and the y-axis shows the log2 ratio of median relative abundance (M, ONT / Illumina) after addition of a pseudocount of 10^− 6^. Only taxa with FDR-adjusted p-values < 0.05 are shown. Positive values indicate higher abundance estimates in ONT, while negative values indicate higher abundance estimates in Illumina

MA plots revealed structured, taxon-specific deviations rather than random noise, suggesting systematic differences in abundance estimates between workflows. Genera that exhibited significantly different RA (FDR < 0.05) estimates in ONT compared to Illumina are reported in the MA plots (Fig. 3). Several taxa showed marked workflow-dependent differences. For example, *Peptacetobacter* (M = 17.00; FDR = 0.0034), *Romboutsia* (M = 15.33; FDR = 0.0034), and *Clostridioides* (M = 14.96; FDR = 0.0038) were relatively enriched in ONT profiles, whereas *Unclassified_Fusobacteriaceae* (M = − 16.86; FDR = 0.0034), *Unclassified_Lachnospiraceae* (M = − 14.32; FDR = 0.0034), and *Collinsella* (M = − 12.48; FDR = 0.0034) were relatively enriched in Illumina profiles.

At the phylum level, the largest workflow-dependent deviations were observed for *Bacillota* (M = 19.59), *Fusobacteriota* (M = 16.13), and *Bacteroidota* (M = 15.47), which showed higher relative abundance estimates in ONT, whereas *Actinobacteriota* showed higher estimates in Illumina (M = − 14.00). At the family level, *Enterobacteriaceae* (M = 13.52), *Enterococcaceae* (M = 9.45), and *Streptococcaceae* (M = 8.67) showed higher estimates in ONT, whereas *Coriobacteriaceae* (M = − 13.57), *Anaeroplasmataceae* (M = − 12.10), and *Peptococcaceae* (M = − 9.85) showed higher estimates in Illumina (Figure S2A–B).

### Age-associated microbiome variation within Illumina and ONT workflows

**Fig. 4:**
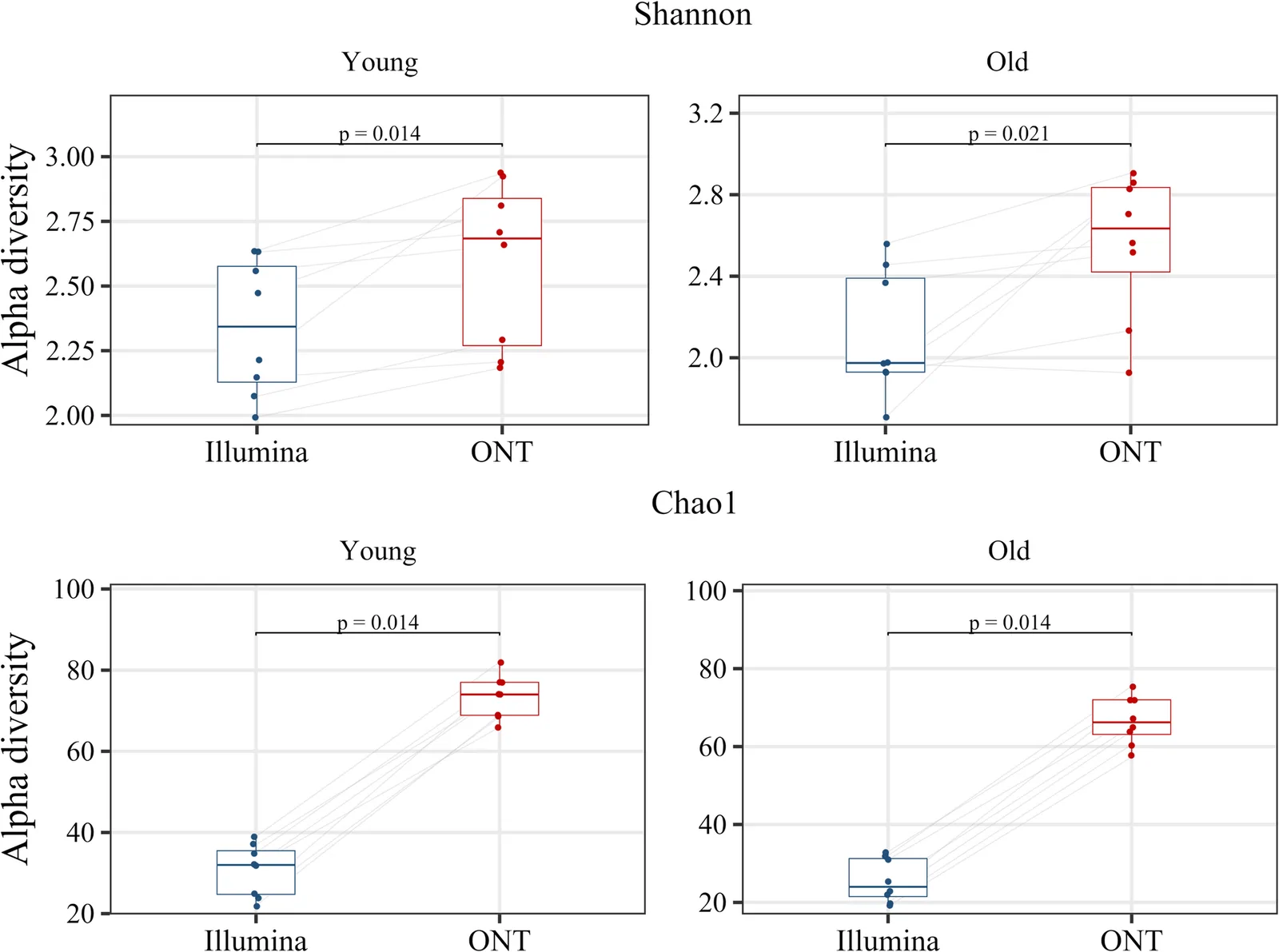
Alpha diversity at the Genus level stratified by age group and sequencing technology. Shannon diversity and Chao1 richness are shown for young and old samples, separately for Illumina and ONT. Boxplots represent the interquartile range, with individual samples overlaid as points. Only Chao1 index differed significantly between old and young dogs for ONT technique (*p* = 0.003)

Before assessing workflow-specific differences, alpha diversity estimates obtained from Illumina and ONT were compared across samples. Chao1 estimates showed a strong positive correlation between workflows (Spearman’s ρ = 0.738, *p* = 0.001), whereas Shannon diversity exhibited only a moderate, non-significant correlation (Spearman’s ρ = 0.432, *p* = 0.094), indicating that agreement between workflows was metric-dependent. Alpha diversity analyses demonstrated that diversity and richness estimates differed between workflows (Fig. 4). At the genus level, ONT data consistently yielded higher Shannon diversity and Chao1 richness compared to Illumina across both age groups (*p* < 0.05). Although age-related patterns were detectable in both datasets, the magnitude of the diversity estimates differed between sequencing workflows.

**Fig. 5:**
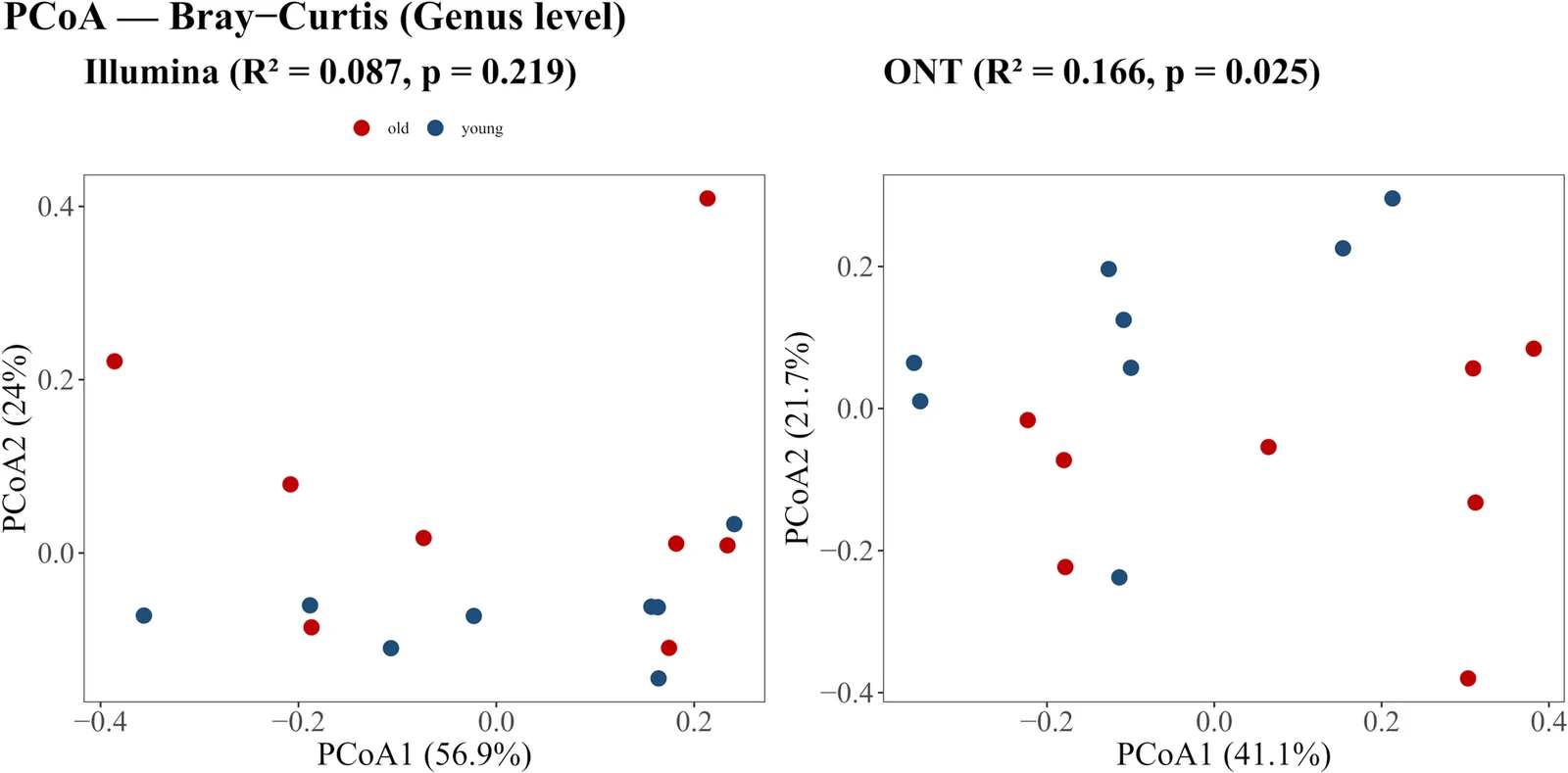
PCoA of Bray–Curtis dissimilarity at the Genus level, stratified by sequencing technology. Ordinations are shown separately for Illumina (left) and ONT (right). Points are coloured by age group. Percentages on axes indicate the proportion of variance explained by each coordinate

Beta diversity analyses performed separately within each sequencing workflow revealed age-associated differences in community composition (Fig. 5). At the genus level, Bray–Curtis PCoA plots showed partial separation between young and old samples in both datasets. PERMANOVA analyses identified nominally significant effects of Age within the ONT dataset at the phylum (R² = 0.204, *p* = 0.038), family (R² = 0.154, *p* = 0.020), and genus (R² = 0.139, *p* = 0.029) levels, whereas no nominally significant effects were detected in the Illumina dataset (Supplementary Table S2). Age-associated community differences were therefore more readily detected in the ONT dataset than in the Illumina dataset.

## Discussion

### Nature of workflow-dependent differences

Our cross-workflow comparison revealed three main patterns of disagreement between Illumina and ONT profiles. Discrepancies between Illumina and ONT involved low-abundance taxa detected by only one workflow. The diagonal bands observed in the Bland–Altman plots (Fig. 2A) indicate that disagreement at low relative abundances is primarily driven by presence/absence discordance rather than quantitative differences. However, even when taxa were detected by both workflows, relative abundance estimates frequently differed. MA plot analyses identified several taxa showing systematic workflow-dependent abundance differences, suggesting that disagreement is not restricted to detection thresholds alone. Only a limited number of phyla showed significant differences between workflows (Figure S2A), whereas a larger number of taxa differed at the family (Figure S2B) and genus (Fig. 3) levels. This pattern suggests that workflow-dependent biases become progressively more evident at finer taxonomic resolution, consistent with known differences in primer coverage, read length, and taxonomic assignment strategies between short- and long-read sequencing workflows (Fouhy et al. 2016; Allali et al. 2017).

These results indicate that taxonomic resolution and quantitative agreement could be considered separately when comparing workflows. Higher taxonomic resolution does not necessarily imply improved quantitative agreement, as substantial differences in relative abundance estimates may still occur between workflows. Differences in primer design, sequencing chemistry, reference databases, and taxonomic assignment algorithms may all contribute to the observed discrepancies and represent important sources of methodological variability (Callahan et al. 2016; Johnson et al. 2019).

### Impact of sequencing workflow on alpha and beta diversity estimates

Alpha diversity metrics, widely used to summarize microbial community complexity, were strongly influenced by sequencing workflow choice. Across both genus and family levels, ONT-based profiles yielded significantly (*p* < 0.05) higher Shannon diversity and Chao1 richness compared to Illumina (Fig. 4). While age-associated trends were broadly preserved across workflows, absolute diversity estimates differed substantially. These findings suggest that alpha diversity metrics are sensitive not only to biological variation but also to the underlying sequencing and analytical framework, as previously discussed in studies addressing bias in diversity estimation (Willis 2019). Consequently, direct comparisons of diversity indices across studies using different sequencing technologies may lead to misleading biological interpretations. In particular, higher richness estimates observed in ONT data may reflect improved detection of low-abundance or closely related taxa enabled by full-length 16S sequencing, but may also be influenced by workflow-specific error structures or taxonomic assignment practices. From a practical perspective, these results indicate that alpha diversity values should not be directly compared across studies employing different sequencing technologies without explicit calibration or methodological harmonization.

By conducting beta diversity analyses separately within each sequencing workflow, we avoided confounding biological signals with cross-workflow compositional differences (Fig. 5). Although age-associated variation was detectable in both datasets, the strength of the statistical signal and the estimated effect sizes differed between workflows (Supplementary Table S2). These findings suggest that broad biological patterns can be recovered using either sequencing approach, while their quantitative representation may vary depending on the analytical workflow employed.

It must be considered that comparisons were performed on RA data, which are inherently compositional and subject to well-known statistical constraints in microbiome analysis (Gloor et al. 2017). Although our analytical framework mitigates some of these issues by using paired designs and within workflow analyses, absolute abundance differences cannot be assessed.

### Limitations

Several limitations should be considered when interpreting these findings. The study was conducted on a relatively small number of dogs, which may limit the generalizability of the observed workflow-dependent patterns. In addition, the comparison involved complete analytical workflows that differed in multiple methodological components, such as primer design, sequencing chemistry, reference databases, and taxonomic assignment pipelines and the contribution of each of these factors to the observed discrepancies cannot be disentangled. Therefore, the present results should be interpreted as a comparison of end-to-end microbiome profiling workflows rather than as an isolated assessment of sequencing technology.

Another limitation is that beta diversity analyses were limited to non-phylogenetic metrics (Bray–Curtis), as reliable phylogenetic trees could not be constructed from aggregated taxonomic tables alone, preventing the evaluation of phylogenetic beta diversity metrics such as UniFrac. Moreover, the Bland–Altman analyses should be interpreted with caution because microbial relative abundance data are compositional and do not fully satisfy the assumptions underlying classical agreement analyses. In addition, the conservative filtering and harmonization strategies adopted prioritized taxonomic reliability over completeness. As a consequence, some low-abundance or workflow-specific taxa could have been excluded, potentially underestimating the extent of cross-workflow divergence.

## Conclusion

Our findings indicate that microbiome profiles generated using different sequencing workflows should not be assumed to be directly interchangeable, particularly at lower taxonomic levels. Workflow-aware interpretation is therefore essential when comparing results across studies, integrating datasets, or conducting meta-analyses.

Although Illumina and ONT produced similar microbiome profiles at higher taxonomic levels, differences emerged at lower taxonomic resolution and in diversity estimates. In particular, taxa uniquely detected by one workflow were generally characterized by low relative abundance, indicating reduced cross-workflow concordance for rare taxa.

Age-associated microbiome patterns were detectable with both sequencing workflows, but their quantitative representation differed depending on the workflow used. These observations suggest that data obtained from different workflows should be interpreted with caution, particularly in meta-analyses.

## Supplementary Information

Below is the link to the electronic supplementary material.

Supplementary Material 1

Supplementary Material 2

Supplementary Material 3

Supplementary Material 4

## Data Availability

Raw data are available at NCBI SRA, accession PRJNA1430067.

## Declarations

### Generative Artificial Intelligence (AI) declaration

ChatGPT Education (OpenAI) was used as an auxiliary tool to assist with code development, graphical design suggestions and language editing. All data processing, statistical analyses, interpretation of results, and final decisions regarding manuscript content were performed and verified by the authors. All figures were generated independently by the authors using R software.
