# Supplementary figures and images for: A comparative analysis of gut microbiome in dogs using short- and long-reads of 16S rRNA sequences reveals workflow-dependent biases
Source: Vet Res Commun. 2026 Jul 17;50(5):463. doi: 10.1007/s11259-026-11407-w (PMC13379502; doi:10.1007/s11259-026-11407-w)

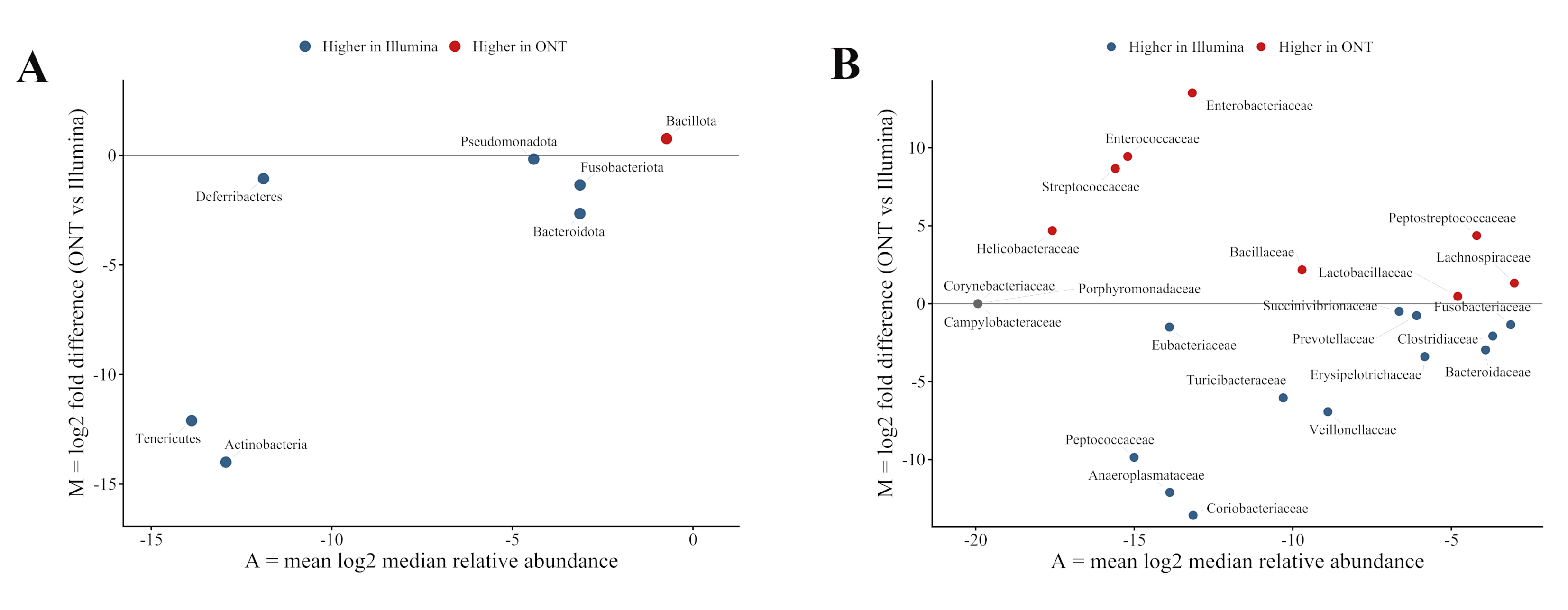

Supplement: Supplementary file 3 — Supplementary Material 3 [file 11259_2026_11407_MOESM3_ESM.tif]

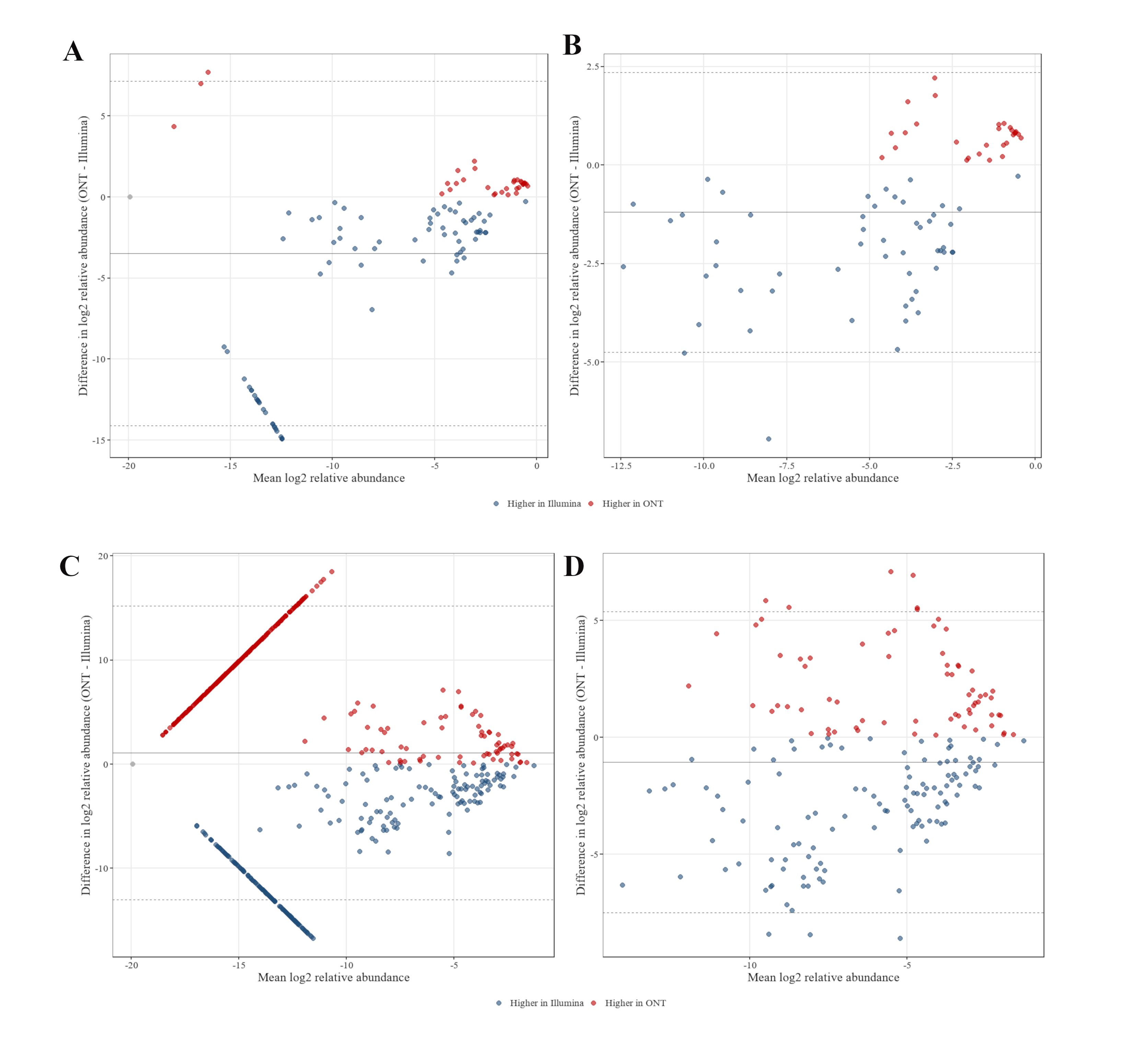

Supplement: Supplementary file 4 — Supplementary Material 4 [file 11259_2026_11407_MOESM4_ESM.tif]
